# Nursing care for patients with cardiorenal syndrome after heart transplantation undergoing continuous renal replacement therapy: A case report and literature review

**DOI:** 10.1097/MD.0000000000043043

**Published:** 2025-06-20

**Authors:** Liyan Zhang, Yi Zhu, Yijing Zhou, Chunyan Chen, Yuanying Xia

**Affiliations:** a Jiaxing Hospital of Traditional Chinese Medicine, Jiaxing, Zhejiang Province, China.

**Keywords:** continuous renal replacement therapy, heart transplantation, personalized care

## Abstract

**Rationale:**

Heart transplantation (HT) represents the optimal treatment for patients with end-stage heart disease. However, it is prone to numerous postoperative complications, among which cardio-renal syndrome (CRS) is particularly serious and carries a high mortality rate. Continuous renal replacement therapy is an essential supportive treatment for these patients, but its efficacy is highly dependent on precise nursing management. Currently, there are few reports on the care of CRS complicating HT both domestically and internationally. This case is presented in this report to provide reference for clinical work.

**Patient concerns:**

This report details the case of a 31-year-old man who underwent an in situ HT due to dilated cardiomyopathy with class IV cardiac function. Following the operation, he developed CRS, which led to oliguria, rapid deterioration of renal function, and cardiac failure.

**Diagnoses:**

Cardiorenal syndrome, chronic kidney disease stage 4, post-dilated cardiomyopathy surgery, HT status, heart function class IV (NYHA classification).

**Interventions:**

This includes implementing a personalized continuous renal replacement therapy (CRRT) program and providing excellent CRRT care; closely monitoring for rejection and the side effects of immunosuppressants; and offering comprehensive psychological support.

**Outcomes:**

After undergoing CRRT for 5 weeks, the patient’s 24-hour urine volume, glomerular filtration rate, and N-terminal brain natriuretic peptide precursor levels stabilized, leading to discharge with improved renal function.

**Lessons:**

The key to a favorable renal function prognosis is the use of CRRT for precise volume management. Careful management of internal jugular vein catheterization is crucial for preventing infections in post-heart transplant patients. Additionally, monitoring the side effects of immunosuppressive drugs and signs of rejection are essential nursing points for patients with cardiorenal syndrome. Providing psychological care in various forms to patients and their families can help improve disease outcomes and ensure long-term efficacy after transplantation.

## 1. Introduction

Heart transplantation (HT) represents the preferred therapeutic option for patients suffering from end-stage heart failure, offering remarkable enhancements in quality of life and substantial extension of survival duration.^[1]^ A multitude of complications can arise during the peri-transplantation period, particularly cardio-renal syndrome (CRS), which presents as both cardiac and renal diseases. In CRS, acute or chronic dysfunction of 1 organ triggers subsequent abnormalities in the other, serving as the primary cause of renal dysfunction in patients with heart failure who previously had normal renal function prior to HT.^[2,3]^

Peri-transplant renal dysfunction serves as an independent risk factor for heightened morbidity and mortality. Research has indicated that between 5% and 29% of HT patients experience renal dysfunction in the peri-transplantation period, necessitating renal replacement therapy.^[4]^

Continuous renal replacement therapy (CRRT) is a technique for blood purification that gradually and consistently eliminates solutes and excess fluid. It offers the benefits of maintaining hemodynamic stability and allowing for precise volume balance control, making it extensively utilized in the management of critically ill patients.^[5]^

On April 17, 2023, our department encountered a case involving post-cardiac transplantation complicated by cardiorenal syndrome. Following aggressive treatment and meticulous care, the patient’s renal function showed significant improvement, leading to a successful hospital discharge. Currently, with a follow-up period of 1 year, the patient’s renal function remains stable. The insights gleaned from this nursing experience are as follows.

## 2. Case presentation

### 2.1. General information

The patient, a 31-year-old male, successfully underwent an in situ HT on March 12, 2023, at an external hospital due to dilated cardiomyopathy and severe cardiac dysfunction classified as NYHA class IV. The surgical procedure was conducted without complications. Post-surgery, the patient’s urine output over a 24-hour period fluctuated between 50 and 125 mL, and he received daily bedside CRRT. Renal function was reassessed on April 16th, revealing a creatinine level of 198 µmol/L. The patient and his family expressed a preference to return to their local facility for the sake of easier follow-up treatment and care. Consequently, the patient was discharged from our hospital on April 16th and subsequently admitted to his local hospital the next day.

Admission examination results: Temperature: 37.1°C, Pulse: 70 beats/min, Respiration: 18 breaths/min, Blood pressure: 107/55 mm Hg. The patient’s consciousness is clear, and the spirit is calm. Physical examination: Heart rhythm is regular, with no heart murmurs detected. Lung sounds are coarse, with a few fine crackles audible. There is no lower limb edema. The right internal jugular vein has a long-term hemodialysis catheter in situ. Additional examinations: Blood tests indicate Potassium: 4.20 mmol/L, Hematocrit: 65 mm/h, N-terminal pro-brain natriuretic peptide: 7613 pg/mL, High-sensitivity troponin-T: 340.4 ng/L, and Creatinine: 416 μmol/L. Cardiac ultrasound reveals a small amount of pericardial effusion; however, the cardiac structure and function appear normal. Admission Diagnosis: Cardiorenal Syndrome, Chronic Kidney Disease Stage 4, Postoperative Dilated Cardiomyopathy, Status Post-Heart Transplant, and Cardiac Function Class IV (per NYHA classification).

### 2.2. Treatment

Upon admission, he underwent electrocardiographic monitoring, oxygen saturation tracking, 24-hour urine output measurement, CRRT, and was administered tacrolimus capsules, enteric-coated mescaline sodium tablets, prednisone tablets for immunosuppression, tolvaptan for sodium conservation and diuresis, ivabradine and dapagliflozin tablets to enhance cardiac function, ferrous succinate to correct anemia, metoprolol delayed-release tablets to decrease ventricular rate, and rosuvastatin calcium tablets for lipid reduction.

CRRT treatment was administered in continuous veno-venous hemofiltration mode, utilizing a Campbell Prismaflex hemofiltration machine equipped with a Prismaflex M150 filter set, which featured an AN69 membrane with a surface area of 1.2 m^2^. Anticoagulation therapy was selected in the form of speedbillin injection, with an initial dosing of 2050 International Units, a maintenance dose of 400 International Units/h, a blood flow rate ranging from 160 to 180 mL/min, a pre-replacement fluid rate of 1000 mL/h combined with a post-replacement fluid rate of 1000 mL/h, a dehydration rate of 0 to 500 mL/h, and a sodium bicarbonate infusion rate of 125 to 145 mL/h.

CRRT was conducted 5 times between April 18th and April 22nd, with each session lasting for 8 hours. The net ultrafiltration volume ranged from 2400 to 2800 mL. On April 24, a reexamination revealed the following results: N-terminal brain natriuretic peptide precursor levels at 2808 pg/mL, high-sensitivity troponin-T levels at 227.5 ng/L, and a cardiac ejection fraction of 73%. Additionally, the patient’s 24-hour urine volume was recorded as 230 mL. Given these findings, renal insufficiency was suspected, prompting a revision of the treatment protocol.

The updated protocol involved switching to continuous veno-venous hemodiafiltration mode, with a dialysis frequency of once every 2 days. The net ultrafiltration volume was adjusted to 400 to 600 mL. After 7 treatments, the patient’s 24-hour urine volume increased to 700 mL, prompting a further adjustment in dialysis frequency to once every 3 days. The remaining dialysis parameters remained unchanged. On May 17, the patient’s 24-hour urine volume was 1300 mL, and the glomerular filtration rate was 20.9 mL/min. On May 18, medical advice was given to suspend CRRT treatment for continuous observation. The changes in some CRRT treatment parameters and related indexes are shown in Table 1.

**Table 1:** Partial CRRT treatment parameters and related indicator changes.

Date	Treatment mode	Water removal (mL)	BNP(pg/mL)	Ejection fraction (%)	Glomerular filtration rate (mL/min)	Next day 24-h urine volume (mL)
April 18th	CVVH	2500	7613	71	8.12	50
April 21st	CVVH	2400	6173	71	9.01	100
April 24th	CVVHDF	600	2808	73	9.65	230
April 30th	CVVHDF	450	4152	72	11.4	480
May 7th	CVVHDF	400	4293	73	13.1	680
May 17th	CVVHDF	600	3910	74	20.9	1300

CRRT = continuous renal replacement therapy, CVVH = continuous veno-venous hemofiltration, CVVHDF = continuous veno-venous hemodiafiltration.

On May 23rd, the patient’s condition improved, and renal function was restored. Consequently, the patient was discharged upon medical advice. During the outpatient clinic visit, the glomerular filtration rate was reviewed at 66.12 mL/min, and the N-terminal brain natriuretic peptide precursor level was 801 pg/mL, indicating a good recovery.

## 3. Nursing

### 3.1. Care of CRRT

#### 3.1.1. Precise capacity assessment and management and implementation of individualized CRRT protocols

Accurately assessing and managing fluid volume in patients can enhance the outcomes of CRRT, mitigate complications, and decrease morbidity and mortality rates.

Precise volume assessment and management: Adopt fine management of fluids, record 24-hour in and out volume, reduce water and sodium intake so as not to aggravate heart failure, and choose water cups and urinals with volume scales for precise recording. During CRRT, 3 levels of volume management were used to record fluid in and out and ultrafiltration volume every hour, and the ultrafiltration volume was adjusted at any time according to precise hemodynamic indexes to prevent the occurrence of insufficient ultrafiltration or excessive ultrafiltration and to maintain organ perfusion and circulating blood volume. Use bedside echocardiography to check the patient’s volume status before starting the machine. In this case, the patient was in the state of HT, and before the patient started CRRT, use bedside echocardiography to check the patient’s volume status, and in consideration of the patient’s low blood pressure and the state of HT, choose the isovolumetric blood-induced on-boarding method: do not exclude the prefilled fluid in the line, and connect and run the dialysis line to the arterial end and the venous end of the indwelling catheter at the same time to maintain the patient’s The patient’s return blood volume remains constant. Echocardiography was also used to assess the recovery of cardiac function and the presence of myocardial ischemia. With CRRT and a series of treatments, the patient’s cardiac ejection fraction increased from 71% on admission to 74% on discharge.

#### 3.1.2. CRRT hemodynamic monitoring and care

Due to the denervation of the donor heart, the transplanted heart has no autonomic innervation, and the heart responds differently to vasoactive drugs than the normal heart, and is more dependent on vasoactive drugs, requiring close hemodynamic monitoring.^[6]^ Hemodynamic indicators such as heart rate, heart rhythm, blood pressure, venous oxygen saturation, hourly urine output and lactate level are closely monitored during CRRT in patients to determine whether patients receive adequate tissue perfusion. Bedside echocardiography and electrocardiography were performed before each CRRT to assess the recovery of cardiac function and the presence of myocardial ischemia. Starting from a low blood flow of 80 mL/min, the blood flow was adjusted to 100 mL/min after 10 minutes, and then slowly increased until the blood flow was 150 mL/min. The dehydration rate was gradually increased from 50 mL/h to the target dehydration rate. In addition, hypothermia is prone to hemodynamic abnormalities and coagulation mechanism disorders,^[7]^ the incidence of hypothermia in CRRT treatment is extremely high accounting for 40% to 59%, and blood temperature and body surface temperature need to be closely monitored.

At the same time to do a good job of patient insulation: pay attention to warmth, adjust the room temperature to 22℃ to 24℃; use the Campbell Prismaflex hemofiltration machine comes with a heater to keep warm, set the heating temperature to 37℃; the replacement fluid is placed in the 37℃ thermostat, ready to use and take; in the course of the treatment, when the patient’s body temperature continues to be lower than 36℃ for more than half an hour, or although it is not up to half an hour, but the patient appeared to have continued to chills, cardiac arrhythmia, hemodynamic instability, etc, the warming blanket to keep warm.

#### 3.1.3. Combined care in CRRT

The joint nursing model with cardiology nurses was adopted as follows: Joint consultation, the nurse of the blood purification center took the initiative to contact the cardiology nurses before the patients were treated with CRRT, and the 2 sides collaborated to carry out a joint consultation on the patients to further analyze their condition. Nursing program development, arranging for cardiology specialist nurses to participate in the CRRT treatment and nursing process, and the cardiology specialist nurses and the specialist nurses of the blood purification center to work together to develop the nursing program of CRRT for patients with HT status. Nursing care in CRRT, cardiology specialist nurses closely monitor the hemodynamic monitoring of patients with cardiac transplantation status, ECG monitoring, timely identification of various high-risk arrhythmias, focusing on the management of fluid intake during the treatment period, and rationally adjusting the infusion rate. In this case, the cardiac rhythm was stable during CRRT treatment, and no abnormality was seen in the electrocardiogram.

#### 3.1.4. Monitoring and care of complications

Common complications of CRRT include air embolism, coagulation abnormalities, electrolyte and acid–base imbalances.^[8]^ Before the machine is connected to the patient, precharge it fully, expel the gas out of the line, tighten each interface of the line, and strictly check the line for air bubbles when the machine alarms to prevent air embolism in the patient.

Coagulation abnormalities are mostly caused by the filter and pipeline factors, the use of anticoagulants in the treatment process, the patient’s own factors and nursing operations, etc. The Prismaflex M150 set filter was selected according to the patient’s condition before the treatment, which has a better filtration efficiency and lower coagulation risk. Two thousand milliliter of physiological saline was used to fully rinse the pipeline and the filter before the treatment and the pipeline was rinsed every 4 hours during the treatment to observe the coagulation of the filter and the pipeline. The filter and tubing were flushed every 4 hours to observe the coagulation situation. The anticoagulant chosen is natriuretic heparin calcium injection, which has the advantages of good anticoagulant effect, high bioavailability and long half-life, etc. Our department regularly monitors the coagulation indexes of the patients and adjusts the dosage of anticoagulant in time. Regular monitoring of blood gas analysis is carried out to understand the patient’s acid–base balance and correct the acid–base imbalance in time. The electrolytes of the patients were measured in the second and sixth hours after boarding the machine, and the formula of replacement fluid was adjusted according to the test results. No complications occurred during the patient’s treatment.

### 3.2. Care of internal jugular vein cannulation

Infection stands as a significant contributor to mortality and complications following HT. Concurrently, the administration of antirejection medications to HT recipients can lead to compromised immune responses.^[9]^ Therefore, meticulous management is imperative in the care of these patients, particularly concerning deep vein catheterization. Medical staff must adhere to stringent aseptic techniques, maintain rigorous hand hygiene, and ensure that nurses take full responsibility for catheter changes. Senior nurses should oversee the entire procedure, promptly addressing any deviations from standard practices. It is essential to inspect the catheter insertion site for signs of bleeding, redness, or swelling. The skin surrounding the catheter orifice should be disinfected with 2% chlorhexidine, with a disinfection area exceeding 10 cm in diameter. Dressings at the catheter site must be kept clean and dry, and 3M sterile dressings should be applied during each catheter change, especially when the dialysis machine is in use. All connections must be made with strict adherence to aseptic protocols to prevent infections.

Before the start of CRRT, it should be judged whether the double lumen tube is fluent, if not fluent with a 20 to 50 mL syringe for high-pressure suction, can not be forced to push saline, so as to avoid coagulation of blood clots into the blood to form a thrombus, pay attention to the placement site of whether there is a blood seepage, if there is a seepage of blood to immediately notify the physician to deal with. Patients are treated in a single room, the air is sterilized before treatment, and instruments and equipment are wiped and sterilized with 0.2% chlorine-containing disinfectant solution before and after use. During the treatment period, the nurse in charge minimized the operation of vascular access to reduce the risk of catheter exposure, disinfected the interface when replacing the replacement fluid, reduced the movement of personnel, prohibited visits during the treatment process, and monitored the blood routine and body temperature after each treatment, and regularly monitored the indicators of infection as well as changes in pathogenicity results.

### 3.3. Care of patients in cardiac transplant status

#### 3.3.1. Observation and care of exclusion reactions

Posttransplant rejection represents a frequent surgical complication and stands as the primary factor contributing to patient mortality. The typical clinical manifestations of heart transplant rejection commonly encompass low-grade fever, fatigue, hypotension, and arrhythmia.^[10]^ Throughout the treatment process, we meticulously monitored changes in heart rate, electrocardiogram readings, and blood pressure. Additionally, body temperature measurements were conducted before, during, and after treatment to track any temperature fluctuations. Prior to the commencement of treatment, bedside Doppler echocardiography was administered to monitor alterations in cardiac output. Notably, no rejection reactions were observed during the patient’s hospital stay.

#### 3.3.2. Immunosuppressant care

To mitigate the risk of organ rejection and simultaneously minimize the risks associated with infections and tumors, HT recipients typically need to take lifelong immunosuppressive medications.^[11]^

A combination of immunosuppressive drugs is often used in clinical practice. The patient was prescribed an oral regimen consisting of tacrolimus capsules, enteric-coated mescaline sodium tablets, and prednisone tablets, with meticulous attention to dosage and frequency. The medical team closely monitored adverse drug reactions and periodically assessed the trough and peak blood concentrations of tacrolimus. Throughout the medication process, particular care was taken to observe any adverse effects of the immunosuppressants, including elevated blood creatinine and aspartate aminotransferase levels, as well as gastrointestinal symptoms such as anorexia, nausea, vomiting, and diarrhea, and hematological changes like leukopenia and fever. By the fifth day, the patient was admitted to the hospital with nausea but without vomiting. The patient was administered pantoprazole sodium enteric-coated tablets, 1 tablet orally 3 times daily as per the doctor’s orders, with favorable outcomes.

### 3.4. Multiform psychological care

The patient had a preexisting focus on maintaining a high quality of life, and both the patient and their family held high expectations for the prognosis of the illness. However, this hospitalization brought about certain negative emotions. On 1 hand, the patient was not well-informed about the CRRT treatment plan and was concerned that the treatment might not be effective, potentially leading to irreversible renal function loss. On the other hand, the patient faced the necessity of long-term medication following HT. Our department acknowledges the patient’s internal anxiety prior to treatment and has taken steps to alleviate it. We familiarize the patient with the environment, distribute educational materials, and explain pertinent disease-related information to bolster the patient’s confidence in overcoming the illness. Given that during treatment the patient’s area is not monitored and is a separate space, the patient and their family may feel uneasy due to the lack of access to treatment information. Therefore, under conditions that the patient’s health permits, we facilitate video calls between the patient and their family members. The responsible nurse explains the CRRT treatment schedule, the process of starting the machine, and the necessary precautions during dialysis. We inform the patient that the dedicated nurses and doctors will be at the bedside throughout the dialysis period. We encourage the patient to listen to music, which helps to alleviate nervousness and anxiety. Through skilled, gentle, and meticulous nursing care, we have gained the patient’s trust, enabling them to actively cooperate with treatment and nursing with the best possible psychological state.

## 4. Conclusion

Cardiorenal syndrome is a prevalent and severe complication following HT, closely associated with morbidity and mortality rates. CRRT offers the benefits of sustaining water, electrolyte, acid–base equilibrium, and internal environment stability, as well as hemodynamic stability. Precise volume management and vigilant hemodynamic monitoring are crucial for a favorable renal prognosis. Moreover, meticulous care during internal jugular vein cannulation is essential to prevent infections in posttransplant patients. It is imperative to monitor the side effects of immunosuppressive medications and to watch for signs of rejection, which are pivotal aspects of patient care for those with combined cardiac and renal issues. Additionally, providing various forms of psychological support to both patients and their families can aid in disease regression and ensure the long-term success of the posttransplantation period.

## 5. Limitations of the study

This study has several limitations. Firstly, it was conducted on a single case, which restricts the generalizability of the findings. Secondly, the study lacked a control group and did not compare different treatments for CRS, which hampers our ability to assess the relative effectiveness of interventions. Additionally, there are relatively few reports on the care of these patients, and it is recommended that healthcare professionals further summarize their care experiences to better guide clinical practice.

## Acknowledgments

The author expresses gratitude for the funding from the Zhejiang Province Traditional Chinese Medicine Science and Technology Project (Project No. 2023ZL174).

## Author contributions

**Methodology:** Yijing Zhou.

**Project administration:** Liyan Zhang, Yijing Zhou, Chunyan Chen, Yuanying Xia.

**Resources:** Yi Zhu, Chunyan Chen.

**Software:** Chunyan Chen.

**Supervision:** Yi Zhu, Yijing Zhou, Chunyan chen, Yuanying Xia.

**Writing – original draft:** Liyan Zhang, Yi Zhu.

**Writing – review & editing:** Liyan Zhang, Yuanying Xia.

## References

[1] Demiralp G Arrigo RT Cassara C Johnson MR. Heart transplantation-postoperative considerations. Crit Care Clin. 2024;40:137–57. 37973350 10.1016/j.ccc.2023.05.004

[2] Zoccali C Zannad F. Refocusing cardio-renal problems: the cardiovascular-kidney-metabolic syndrome and the chronic cardiovascular-kidney disorder. Nephrol Dial Transplant. 2024;39:1378–80. 38599634 10.1093/ndt/gfae086

[3] Lee S Ban TH Park HS. Role of renal replacement therapy during the peri-transplant period of heart transplantation. Ann Transplant. 2020;25:e925648. 33230094 10.12659/AOT.925648 PMC7697654

[4] Kumar N Fitzsimons MG Bardia A Dalia AA. Renal replacement therapy after heart transplantation: symptom or syndromic? J Cardiothorac Vasc Anesth. 2024;38:1823–4. 38876813 10.1053/j.jvca.2024.04.001

[5] Rachoin JS Weisberg LS. Renal replacement therapy in the ICU. Crit Care Med. 2019;47:715–21. 30768442 10.1097/CCM.0000000000003701

[6] Teixeira JP Zeidman A Beaubien-Souligny W. Proceedings of the 2022 UAB CRRT Academy: non-invasive hemodynamic monitoring to guide fluid removal with CRRT and proliferation of extracorporeal blood purification devices. Blood Purif. 2023;52:857–79. 37742622 10.1159/000533573

[7] Bergman L Lundbye JB. Acid-base optimization during hypothermia. Best Pract Res Clin Anaesthesiol. 2015;29:465–70. 26670817 10.1016/j.bpa.2015.09.005

[8] Gautam SC Lim J Jaar BG. Complications associated with continuous RRT. Kidney360. 2022;3:1980–90. 36514412 10.34067/KID.0000792022 PMC9717642

[9] Benatti RD Oliveira GH Bacal F. Heart transplantation for chagas cardiomyopathy. J Heart Lung Transplant. 2017;36:597–603. 28284779 10.1016/j.healun.2017.02.006

[10] Li H Chen Y Jin Q. Noninvasive radionuclide molecular imaging of the CD4-Positive T Lymphocytes in acute cardiac rejection. Mol Pharm. 2021;18:1317–26. 33506680 10.1021/acs.molpharmaceut.0c01155

[11] Roberts MB Fishman JA. Immunosuppressive agents and infectious risk in transplantation: managing the “Net State of Immunosuppression.”. Clin Infect Dis. 2021;73:e1302–17. 32803228 10.1093/cid/ciaa1189 PMC8561260

